# Bayesian Spatiotemporal Modeling of Routinely Collected Data to Assess the Effect of Health Programs in Malaria Incidence During Pregnancy in Burkina Faso

**DOI:** 10.1038/s41598-020-58899-3

**Published:** 2020-02-14

**Authors:** Toussaint Rouamba, Sekou Samadoulougou, Halidou Tinto, Victor A. Alegana, Fati Kirakoya-Samadoulougou

**Affiliations:** 1 Clinical Research Unit of Nanoro, Institute for Research in Health Sciences, National Center for Scientific and Technological Research, 528, Avenue Kumda-Yoore, BP 218 Ouagadougou CMS 11, Ouagadougou, Burkina Faso; 2 Center for research in epidemiology, Biostatistics and Clinical Research, School of Public Health, University libre de Bruxelles (ULB), Route de Lennik, 808 B-1070 Bruxelles, Brussels, Belgium; 3 Centre for Research on Planning and Development (CRAD), Laval University, Quebec, G1V 0A6 Canada; 4 Evaluation Platform on Obesity Prevention, Quebec Heart and Lung Institute, Quebec, G1V 4G5 Canada; 5 Kenya Medical Research Institute - Wellcome Trust Research Programme, Nairobi, Kenya; 6 Geography and Environmental Science, University of Southampton, SO17 1BJ Southampton, UK

**Keywords:** Malaria, Epidemiology, Epidemiology

## Abstract

Control of malaria in pregnancy (MiP) remains a major challenge in Burkina Faso. Surveillance of the burden due to MiP based on routinely collected data at a fine-scale level, followed by an appropriate analysis and interpretation, may be crucial for evaluating and improving the effectiveness of existing control measures. We described the spatio-temporal dynamics of MiP at the community-level and assessed health program effects, mainly community-based health promotion, results-based financing, and intermittent-preventive-treatment with sulphadoxine-pyrimethamine (IPTp-SP). Community-aggregated monthly MiP cases were downloaded from Health Management Information System and combined with covariates from other sources. The MiP spatio-temporal pattern was decomposed into three components: overall spatial and temporal trends and space-time interaction. Bayesian hierarchical spatio-temporal Poisson models were used to fit the MiP incidence rate and assess health program effects. The overall annual incidence increased between 2015 and 2017. The findings reveal spatio-temporal heterogenicity throughout the year, which peaked during rainy season. From the model without covariates, 96 communities located mainly in the Cascades, South-West, Center-West, Center-East, and Eastern regions, exhibited significant relative-risk levels. The combined effect (significant reducing effect) of RBF, health promotion and IPTp-SP strategies was greatest in 17.7% (17/96) of high burden malaria communities. Despite intensification of control efforts, MiP remains high at the community-scale. The provided risk maps are useful tools for highlighting areas where interventions should be optimized, particularly in high-risk communities.

## Introduction

In Sub-Saharan Africa (SSA) countries, the weak health system impacts the effective delivery of health care, which contributes to a high disease burden^1^. In Burkina Faso, due to the technical and financial support from development partners, efforts have been made to strengthen the health system and improve health coverage. Despite some progress, malaria remains a key public health concern among vulnerable populations, especially children aged under five years and pregnant women^2–5^. Malaria infection during pregnancy can contribute to the risk of miscarriage, stillbirth, premature birth, low birth weight, fetal loss in addition to maternal death^6^. It also contributes to several early childhood impairment^7^. Pregnant women represent an easily accessible population due to their attendance at health facilities for antenatal care (ANC) services and were recently suggested as a credible population for surveillance of the malaria burden at the community level^8^. Recently, studies would suggest that MiP burden is correlated with malaria burden in other population (children)^7,9^ and model-based malaria endemicity measures^10^.

Currently, in malaria endemic countries, malaria control measures for pregnant women include the use of long-lasting insecticide-treated nets (LLINs), intermittent preventive treatment with sulphadoxine-pyrimethamine (IPTp-SP), case management and surveillance through routinely-collected data^11,12^. In Burkina Faso context, the high rate of ANC coverage (83%) during pregnancy^5,13^ provides an opportunity to implement strategies to reduce the malaria in pregnancy (MiP) burden and also helps to reduce the reservoir of malaria transmission in the community. To enhance the efficiency of existing control measures and improve the local planning and allocation of scarce resources, various strategies have been recently introduced to strengthen community-based health promotion, with a focus on primary prevention^14^. This initiative was implemented in 2015 and was mainly aimed at the decentralization of health care through strengthening the technical and operational capacities of communities to improve their own health, developing preventive health care services, and addressing health care issues like malaria with proximity and mass communication activities. A results-based financing (RBF) strategy was also implemented in health facilities to improve health service quality and use, which could generally enhance well-being and improve health indicators, and specifically improve maternal and child health^15^.

Although all these efforts have led to a modest reduction in maternal deaths related to severe MiP, the number of MiP cases remains high^5^. Currently, to the best of our knowledge, little research has been conducted on the national spatio-temporal distribution of the malaria burden during pregnancy at the administrative fine-scale (community-level) in Burkina Faso. Hierarchical Bayesian conditional auto-regressive (CAR) analysis^16^ is used to quantify patterns of malaria at small areal levels via smoothing. These estimates can assist with the implementation of the National Malaria Program (NMP), health-district managers, and municipal decision-makers in planning and optimizing interventions during pregnancy.

We aimed to analyze Burkina Faso’s monthly routinely collected malaria data at the community level to better understand the spatio-temporal pattern of malaria incidence during pregnancy by producing spatio-temporal dynamic maps. Our secondary objective was to evaluate the effect of health program interventions (community-based health promotion, RBF and IPTp-SP) on MiP at community-level adjusted for meteorological, socioeconomic, and contextual factors. We hypothesized that health program interventions, especially community-based health promotion projects and RBF programs, have considerably improved the management of malaria cases during pregnancy through an increase in the number of malaria cases diagnosed.

## Methods

### Settings

**Figure 1:**
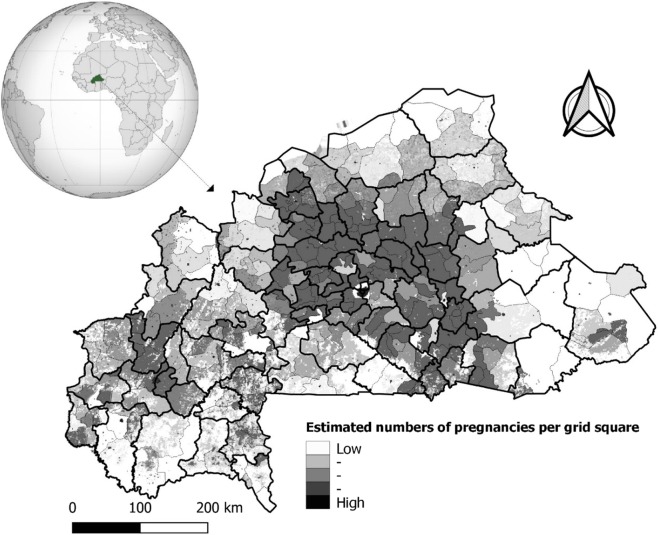
Map of Burkina Faso with small administrative divisions nested to health-districts areas and Spatial pattern of estimated pregnancies per grid square. Black bold lines correspond to limits of the health-districts and gray lines in each health-district correspond to limits of communities. Maps created by Toussaint Rouamba *et al*., 2019. Source: The shapefile was obtained from the “Base Nationale de Découpage du territoire” of Burkina Faso (BNDT, 2006). The estimates of numbers of pregnancies per grid square were obtained from WorldPop (www.worldpop.org - School of Geography and Environmental Science, University of Southampton). The globe (name: orthographic map of Burkina Faso) used in this Figure is created by Martin23230 and freely available at https://commons.wikimedia.org/wiki/File:Burkina_Faso_(orthographic_projection).svg under CC BY-SA 3.0 License (https://creativecommons.org/licenses/by-sa/3.0/deed.en), which lets others remix, tweak, and build upon their work even for commercial purposes, as long as they credit author and license their new creations under the identical terms.

Burkina Faso is located between latitudes 9° and 15° N and longitudes 6° W and 3° E. It is a country in the center of Western Africa with a surface area of 274,200 km^2^. It has six neighboring countries: Togo and Ghana to the south, Mali to the north, Niger to the east, Côte d’Ivoire to the southwest, and Benin to the southeast^17^. In 2017, the population was estimated to be about 20 million with 51.7% women. The population distribution is concentrated in the central and southern parts of the country, with the east, north, and southwest regions being the least populated^5,18^. The country is divided into 13 administrative regions. These regions encompass 45 provinces and 351 communities or departments. The planning of health resource location is allocated at the district-level (*n* = 70) with micro stratification at community/department level (*n* = 351)^19^. Figure 1 shows a map of Burkina Faso with small administrative divisions (351 communities) nested in health-district areas. In 2017, there were 70 health district areas, and malaria represented the most common cause of outpatient attendance (43.5%) in peripheral health facilities.

### Data sources and data description

#### Data from district health information system: malaria in pregnancy cases and IPTp-SP data

Currently, malaria data for routine surveillance in Burkina Faso can be obtained using two methods: from the National Epidemiological Surveillance System, known as “Telegram Official Hebdomadary Letter”, which requires each health facilities to provide weekly reports on 11 diseases including malaria to their respective health districts to allow for early warnings; the national database known as District Health Information System 2 (DHIS2). These data are collected routinely by health facilities in the framework of the NMP, and reported cases are then assembled, checked, and validated by the health-district staff before being entered electronically into the DHIS2 database. The DHIS2 data are subsequently checked and validated first by the Regional Health Directorate and then by the Sectoral Statistics Directorate of the Ministry of Health before any use. The main purpose of the latter database is to facilitate the management of malaria diagnosis and treatment materials. Therefore, this database generates regular monthly reports on the malaria case numbers (disaggregated to consider children under five, pregnant women, and others), the antimalarial drug stocks, as well as the number of LLINs and IPTp-SP distributed to pregnant women. These monthly data are available in aggregate form (regional, province, health-district, or community level) and encompass all health facilities nationally (public and private). The national routine data quality assessment for malaria data showed that the completion and promptitude rate was estimated to be 94.7%, 99.95%, and 99.6% in 2015, 2016, and 2017, respectively, whereas the concordance index was estimated to be 75.9%, 73.0%, and 72.1% for the same periods, respectively.

Since routine weekly malaria case data are not yet entered into DHIS2, we used MiP cases (count data) reported in the national malaria database (https://burkina.dhis2.org) between January 2015 and December 2017. Likewise, the data regarding the proportion of women who received at least three doses of IPTp-SP during the entire course of pregnancy for the same period were also downloaded.

These downloaded data were aggregated by month and by community and included malaria cases (diagnosed by RDT according to the national guidelines) among pregnant women attending health facilities, regardless of age.

#### Potential determinants of MiP data

In addition to the proportion of pregnant women who received at least three doses of IPTp-SP, meteorological variables, the poverty index, type of community (rural or urban), and proportion of women under 20 years old, which are known to influence the distribution of malaria, were selected as covariates for modeling the malaria incidence rate^20–24^.

Meteorological variables for each community, such as the average monthly temperature (°C) and the total monthly rainfall (mm) were obtained using data retrieved from WorldClim at a spatial resolution of 1 km (http://worldclim.org/version2).

The poverty index of each community was obtained from the final 2014 report on poverty and inequality mapping in Burkina Faso^25^. The latter is an indicator of socioeconomic disadvantage and mainly includes the following variables: proportion of households without electricity, without access to drinking water, proportion of unemployed people in the workforce population, housing building materials, the number of inhabitants per household, and the possession of capital goods. The index was built using the small area estimation method developed by the ELL^26^.

The proportion of women under 20 years of age aggregated by year and by community was obtained from the projections of the Burkina Faso National Institute of Statistics and Demography (INSD)^27^.

Variables related to the presence of a health program in a community, mainly RBF implemented in 2014 and the project promoting health in 130 communities implemented in 2015, were obtained through the Minster of Health. These variables were used as binary variables, with “Yes” as the response if the health program was present in the community and “No” otherwise. The geographic distribution of these two health programs is presented in Fig. S1.

#### Number of pregnancies at risk per community

The community administrative boundaries were obtained from the Global Administrative Areas database (https://gadm.org/download_country_v3.html). A unique identification code was used to link each piece of community aggregated data with the shapefile. The arrondissements (administrative units) of the two main capitals, Ouagadougou and Bobo-Dioulasso, were grouped into contiguous community areas.

The estimated number of pregnant women at risk of malaria in the 2015 was downloaded from Afripop through Worldpop (Fig. 1)^17^ in raster format. Then the number of pregnancies for each community was extracted using the “*raster*” package implemented in R software (R Development Core Team, R Foundation for Statistical Computing, Vienna, Austria). This estimation of number of pregnancies was derived from different datasets on population distribution, age structure, and fertility rates, along with estimates for stillbirths, miscarriages, abortions, and national estimates for live births^17^. The original pregnancy population grid (1 km²) produced for 2015 from Afripop was projected in 2016 and 2017 based on projections from INSD^27^. In the present study, all pregnancies across the country were considered to be at risk of malaria.

### Statistical analysis

#### Crude incidence rate, crude standardized morbidity ratio of MiP, and cross-correlation between meteorological variables

The crude monthly incidence rate of MiP for each community was computed using the following formula: $Incidence\,rat{e}_{kt}={Y}_{kt}/{P}_{kt}$, where ${Y}_{kt}$ is the observed number of MiP cases and ${P}_{kt}$ is the number of pregnant women at risk in community *k* in month *t*. Then, the monthly incidence rate was transformed into a time series to explore seasonal patterns and temporal trends in MiP and to assess temporal correlations with meteorological time series. Therefore, a multiplicative decomposition (seasonal pattern, temporal trend, and remainder) as well as an autocorrelogram were applied to the MiP incidence rate. Lag times between time series of the monthly MiP incidence rate and both monthly rainfall and the monthly average temperature were determined using a cross-correlation function.

To consider the excess number of MiP cases in community *k* at time *t*, and to quantify the increased or decreased incidence of cases with respect to the population of pregnant women, a crude standardized morbidity ratio (SMR) was estimated as $SM{R}_{kt}={Y}_{kt}/{E}_{kt}$, where ${E}_{kt}$ is the expected number of MiP cases in community *k* across month *t*. The expected number of MiP cases in community *k* was computed as ${E}_{kt}=\lambda {P}_{kt}$, where *λ* is the overall incidence rate equal to ${\sum }^{}{Y}_{k}/{\sum }^{}{P}_{k}$.

#### Assessing the presence of spatial autocorrelation in the incidence of MiP

The spatial autocorrelation of MiP cases was assessed by computing residuals from a preliminary non-spatial over-dispersed Poisson log-linear model that incorporates covariate effects at the community level. The covariates we used were factors that may influence the coverage of maternal health services (health programs and IPTp-SP) and factors that influence the spatio-temporal dynamics of malaria, as commonly described in previous malaria studies in SSA countries (meteorological, poverty, location, age structure)^20–24^.

The number of MiP cases within a community *k* at time *t*, noted as ${Y}_{kt}$, was the response variable, and we assumed that ${Y}_{kt} \sim Poisson\,({E}_{kt}{\theta }_{kt})$, where ${\theta }_{kt}$ is the risk relative to ${E}_{kt}$, which is the expected number of cases in a community *k* at month *t*. To quantify the presence of spatial autocorrelation in residuals from this non-spatial model, Moran’s *I* statistic was computed for each month, and permutation tests were performed for each month of data separately using Monte-Carlo simulations with 10,000 replications^28^. To compute Moran’s *I* statistic, a binary neighborhood matrix *W* based on the border sharing of communities was built. This neighborhood matrix was a 351 × 351 symmetrical matrix at community level. Two communities were neighbors if they shared a common boundary, so ${w}_{ji}$ was assigned a value of 1 when ${S}_{j}$ and ${S}_{i}$ communities shared a common border, or 0 otherwise.

#### Bayesian spatio-temporal modeling of incidence rates of MiP

Bayesian spatio-temporal modeling of MiP cases was performed using the CARBayesST package developed by Lee *et al*.^29^. Spatial and temporal autocorrelations (random effects) were modeled via the conditional autoregressive (CAR) method proposed by Leroux et al.^30^. Each set of random effects is mean-centered. Thus:

1 $${Y}_{kt} \sim Poisson\,({\mu }_{kt})$$

2 $${ln}({\mu }_{kt})={X}_{kt}^{T}\beta +{O}_{kt}+{\psi }_{kt}$$

where ${\mu }_{kt}$ is the expectation (mean) of the observed MiP case count ${Y}_{kt}$, ${X}_{kt}$ are explanatory space–time covariates associated with the mean process with $\beta$ coefficients, ${O}_{kt}$ is a space–time vector of known offset, and ${{\rm{\psi }}}_{kt}$ is a set of spatio-temporally autocorrelated random effects for community *k* and time period *t*. The proposed model decomposes the spatio-temporal random effects into 3 components^31^ as follow:

3 $${\psi }_{kt}={\phi }_{k}+{\delta }_{t}+{\gamma }_{kt}$$

where ${\phi }_{k}$ and ${\delta }_{t}$ is the overall spatial and temporal main effects respectively, and ${\gamma }_{kt}$, the space-time interaction.

The first component captures the overall spatial random effect common to all time periods after adjusting for covariate effects, denoted by $\phi =({\phi }_{1},\ldots ,{\phi }_{351})$. In our study, the spatial relationships between communities were binary neighborhood matrixes *W*, as defined above.

4 $${\phi }_{k}|{{\boldsymbol{\phi }}}_{-k},\,{\boldsymbol{W}} \sim N(\frac{\rho S{\sum }_{j=1}^{K}{w}_{kj}{\phi }_{j}}{\rho S\,{\sum }_{j=1}^{K}{w}_{kj}+1-\rho S},\frac{{\tau }_{S}^{2}}{\rho S\,{\sum }_{j=1}^{K}{w}_{kj}+1-\rho S})$$

The second component is a temporal random effect that captures the overall temporal trend common to all communities, denoted by $\delta =({\delta }_{1},\ldots ,{\delta }_{36})$.

5 $${\delta }_{t}|{{\boldsymbol{\delta }}}_{-t},\,{\boldsymbol{D}} \sim N(\frac{\rho T{\sum }_{j=1}^{N}{d}_{tj}{\delta }_{j}}{\rho T{\sum }_{j=1}^{N}{d}_{tj}+1-\rho T},\,\frac{{\tau }_{T}^{2}}{\rho T{\sum }_{j=1}^{N}{d}_{tj}+1-\rho T})$$

In our study, temporal relationships between MiP cases were determined using an adjacency weights matrix, a binary $N\times N$ (*N* = 1, …, 36) temporal neighborhood matrix $D={d}_{tj}$, where ${d}_{tj}=1$ is defined if $|j-t|=1$, and ${d}_{tj}=0$ otherwise.

The third component is a set of independent space–time effects interactions, denoted by $\gamma =({\gamma }_{1},\ldots ,{\gamma }_{351\times 36})$.

6 $${\gamma }_{kt} \sim N(0,\,{\tau }_{I}^{2})$$

$${\tau }_{S}^{2},\,{\tau }_{T}^{2},\,{\tau }_{I}^{2} \sim Inverse-Gamma(1,0.01),$$

where *τ*² represents variance in spatial, temporal, and space–time random effects.

$$\rho S,\,\rho T \sim Uniform(0,\,1)$$

where $\rho S$ and $\rho T$ are spatial and temporal dependence parameters, respectively, that govern the strength of spatial and temporal autocorrelations.

Models goodness of fit were compared using the deviance information criterion (DIC)^32^. Spatio-temporal model 1 was fitted without covariates (null model); models 2 and 3 included meteorological covariates (temperature, two-months lag for rainfall), socio-economic factors and contextual variables (poverty-index and locality), the proportion of women who received at least three doses of IPTp-SP, and the presence of a health program in a community, mainly RBF for Health and the Health Promotion in 130 Communities project. Models 2 and 3 were adjusted for seasonality and proportion of women under 20 years of age to consider the high risk of malaria in primigravida. In Burkina Faso, the median age of women at first childbirth (first delivery) is estimated to be 19.5 years^33^. Model 2 was implemented without a space–time interaction, whereas model 3 included a space–time interaction. All models were fitted in a hierarchical Bayesian spatio-temporal setting using Markov chain Monte Carlo (MCMC) simulation.

The posterior distributions of model parameters were obtained on 50,000 MCMC samples generated from a single Markov chain, which was executed for 550,000 iterations with a burn-in period of 50,000 and then thinned by 10 to reduce Markov chain autocorrelation. Convergence was monitored both graphically and using the convergence diagnostics proposed by Geweke^34^.

### Ethic

The data used in this analysis are health facility aggregated data reported in the District Health Information System 2 (DHIS2) and provided by the Directorate General of Studies and Sectoral Statistics of the Ministry of Health. A waiver has been granted by the National Ethics Committee (“Comité d’Ehtique pour la Recherche en Sante (CERS)” of Burkina Faso (N°2019-79/MS/MESRSI/CERS du 19 Jul 2019). The data analysis was carried out at Community level with no reference to individual level identification particulars.

## Results

### Incidence of malaria in pregnancy and cross-correlation with meteorological time series

**Figure 2:**
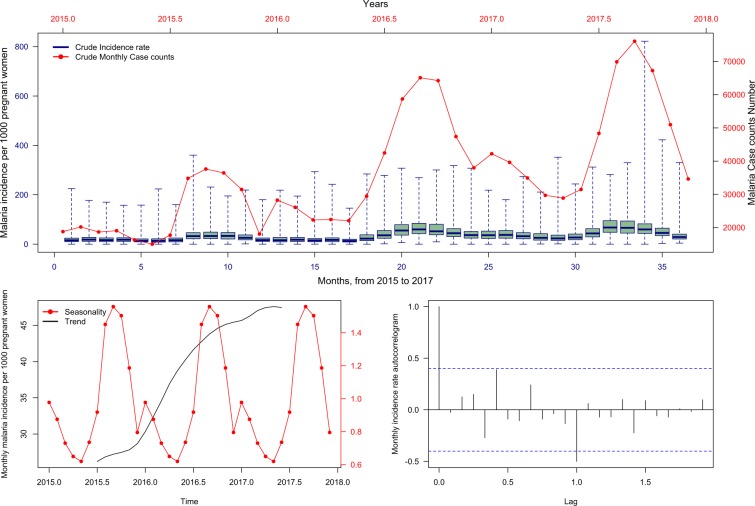
Malaria in pregnancy monthly incidence per 1000 and case counts during the study period (2015–2017). The top layer shows the temporal pattern in original time series (reported cases). The bottom left layer shows the decomposed components into seasonal and trend component. The bottom right layer shows decomposed remainder component.

Nationally, the overall annually observed number of reported MiP cases at the community-level increased between 2015 and 2017. The annual MiP crude cumulative incidences were estimated at 310, 493, and 567 cases per 1000 pregnant women per year for 2015, 2016, and 2017, respectively. The time-series decompositions of the crude monthly MiP incidence rate over the study period peaked between August and November (Fig. 2).

**Figure 3:**
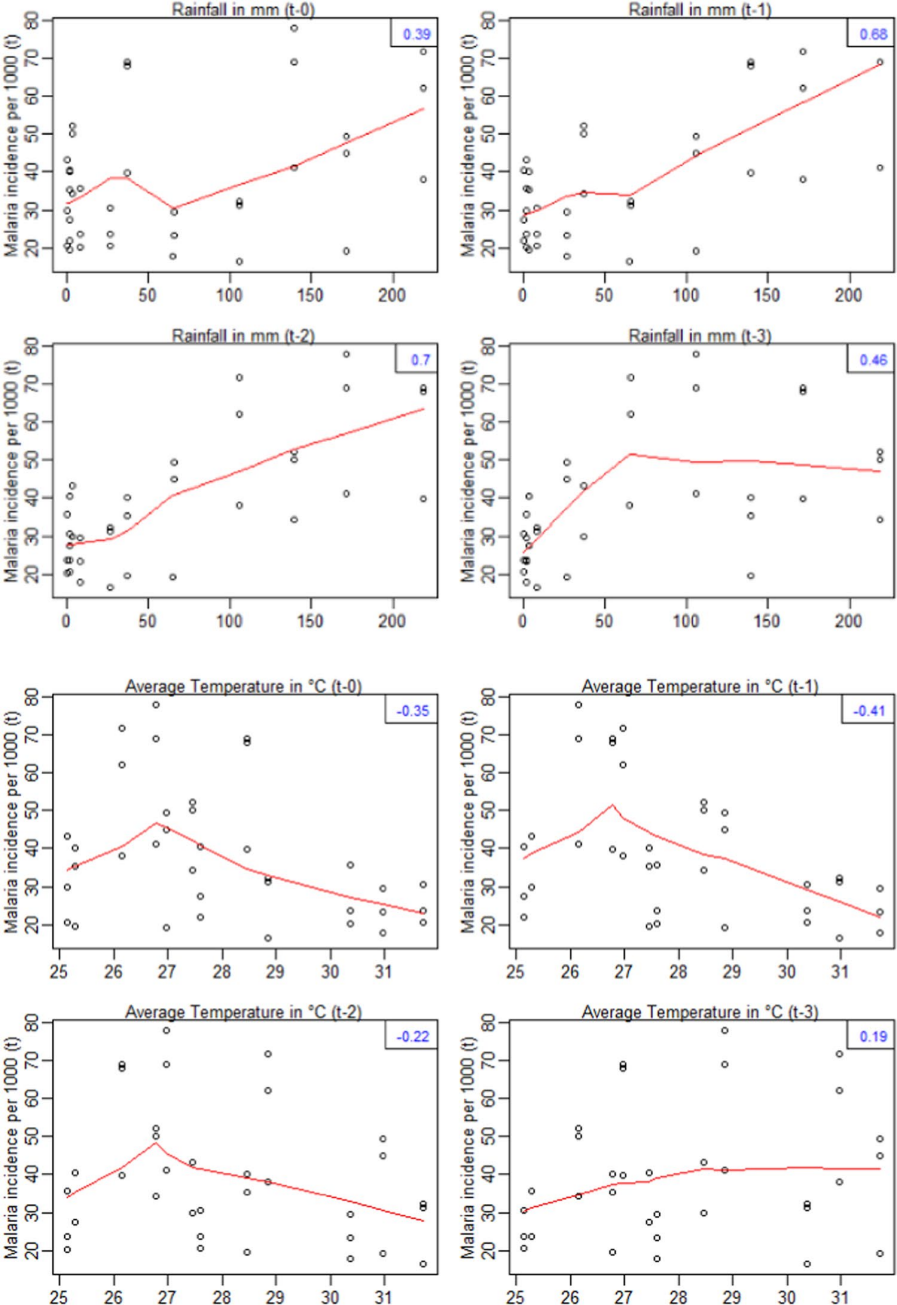
Relationship (cross-correlation) between malaria in pregnancy incidence rate and meteorological variables. The red curves represent smooth relationships of incidence rate according to the meteorological variables and the lag-times. Numbers in blue (top right of each frame) represent the values of the correlation. The first four figures at the top represents the cross-correlation between monthly malaria incidence rate and monthly rainfalls. The last four figures represent the cross-correlation between monthly malaria incidence rate and monthly average temperature.

Rainfall and temperature time series are presented in Fig. S2. Significant and strong positive correlations were identified for MiP time series lagging one to three months behind precipitations. This suggested that the MiP peaks of a specific month were influenced by the rainfalls occurring one to three months ago. However, MiP time series were significantly and negatively correlated with zero and one month lagging the temperature (Fig. 3).

Nationally, the distribution of the burden of MiP in terms of absolute case numbers (Fig. S3), incidence rates (Fig. S4) and standardized morbidity ratio (Fig. S5). Cases of MiP occurred in each community regardless of the month and year. This was particularly evident for the communities located in east, central-east, Cascades, and south-west regions.

### Bayesian results for spatio-temporal changes in MiP incidence

The monthly Moran’s *I* statistics obtained from the residuals of a non-spatial multivariable over-dispersed Poisson log-linear model are presented in Table S1. Moran’s *I* values ranged from 0.05 to 0.23 suggesting autocorrelation in residuals. Autocorrelation of residuals suggested the presence of spatial heterogeneity unaccounted for, or covariates (probably clustered in the landscape) that were not included in the model. In terms of the lowest DIC values, the Bayesian spatio-temporal multivariable analysis from model 3 with interaction was the best model. The convergence plots are presented in Fig. S6.

The spatial and temporal dependence parameters from the Bayesian null model (*ρS* = 0.612; *ρT* = 0.942) and model 3 (*ρS* = 0.573; *ρT* = 0.868), respectively are presented Table 1.

**Table 1:** Median of posterior regression coefficients and 95% credible interval from hierarchical Bayesian spatio-temporal modeling of malaria in pregnancy reported by month and communities, Burkina Faso, 2015–2017.

Indicator	Null model	Model 2^†^	Model 3^†^
	Coef (95% CrI)	Coef (95% CrI)	Coef (95% CrI)
**Fixed effects**			
Intercept^†^	0.2247 (0.245, 0.249)	0.111 (0.070, 0179)	0.059 (0.031, 0.108)
Proportion of women who received at least three doses of IPTp-SP^†^		1.000 (0.996, 1.003)	0.999 (0.998, 1.002)
**Meteorological variables**^†^			
Rainfall with 2 months lag		1.000 (0.999, 1.001)	1.016 (1.013, 1.019)
Temperature (°Celsius)		0.999 (0.998, 1.001)	0.999 (0.999, 1.001)
Socio-economic and contextual variables^†^			
Poverty-index		1.001 (0.997, 1.006)	0.999 (0.993, 1.005)
Living in rural area		1	1
Living in urban area		1.108 (1.040, 1.117)	0.929 (0.679, 1.216)
Health promotion project^†^			
No		1	1
Yes		1.010 (0.968, 1.057)	1.009 (0.966, 1.052)
Result-based financing^†^			
No		1	1
Yes		1.047 (0.988, 1.110)	1.046 (0.985, 1.111)
**Spatio-temporal random effects**^**‡**^			
Median spatial random effect $({\phi }_{k})$	0.000 (−0.118, 0.108)	0.020 (−0.103, 0.140)	0.019 (−0.105, 0.141)
Median of monthly trend $({\delta }_{t})$	0.012 (−0.031, 0.051)	0.012 (−0.040, 0.062)	0.014 (−0.060, 0.089)
Median of space-time interactions $({\gamma }_{kt})$			0.000 (−0.058, 0.058)
**Spatio-temporal random effects variances**^‡^			
Spatial random effects variance $({\tau }_{S}^{2})$	0.108 (0.082, 0.140)	0.104 (0.079, 0.135)	0.099 (0.074, 0.130)
Temporal random effects variance $({\tau }_{T}^{2})$	0.018 (0.011, 0.031)	0.004 (0.003, 0.008)	0.016 (0.009, 0.029)
Space-time random effects variance $({\tau }_{I}^{2})$			0.0008 (0.0006, 0.0013)
**Spatial and temporal dependence**^**‡**^			
Spatial dependence (*ρS*)	0.612 (0.399, 0.838)	0.613 (0.397, 0.843)	0.573 (0.356, 0.814)
Temporal dependence (*ρT*)	0.942 (0.805, 0.993)	0.975 (0.940, 0.997)	0.868 (0.614, 0.979)
**DIC**	57154	57135	57020

^**†**^Coefficient on antilog scale; ^**‡**^Coefficient on log scale, ^†^adjusted for seasonality and proportion of women under 20 years of age

Note: *CrI:* credible interval, *DIC*: deviation information criteria, IPTp: Intermittent preventive treatment during pregnancy, SP: sulphadoxine-pyrimethamine.

**Figure 4:**
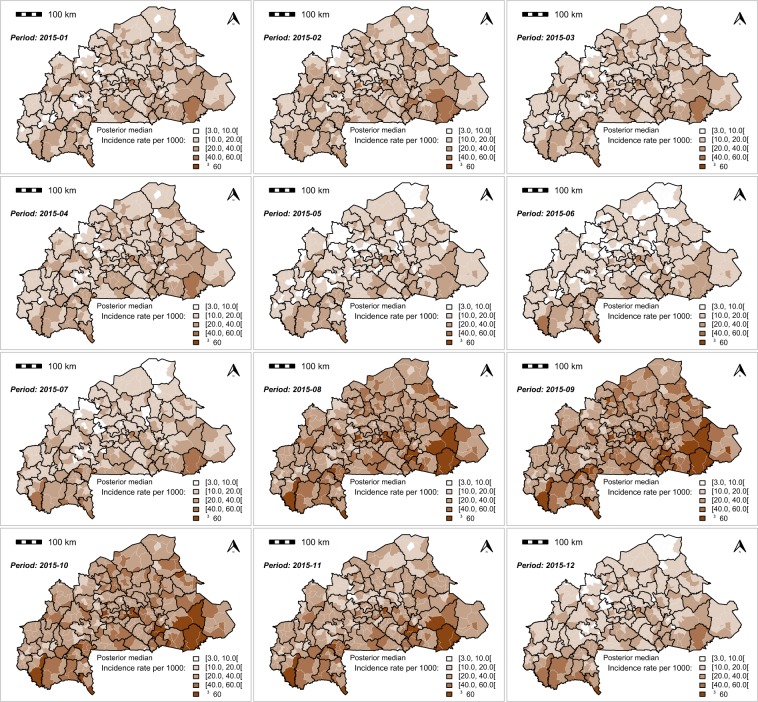
Spatio-temporal dynamic of malaria in pregnancy (MiP) monthly incidence as a rate (per 1000) of fitted values (based on posterior medians) and total number of pregnant women at risk per community for Year 2015. Source: The shapefile was obtained from the “Base Nationale de Découpage du territoire” of Burkina Faso (BNDT, 2006). The malaria in pregnancy data were obtained from the National Malaria Control Program of Burkina Faso, downloaded from the District Health Information System 2. Maps created by Toussaint Rouamba *et al*., 2019.

**Figure 5:**
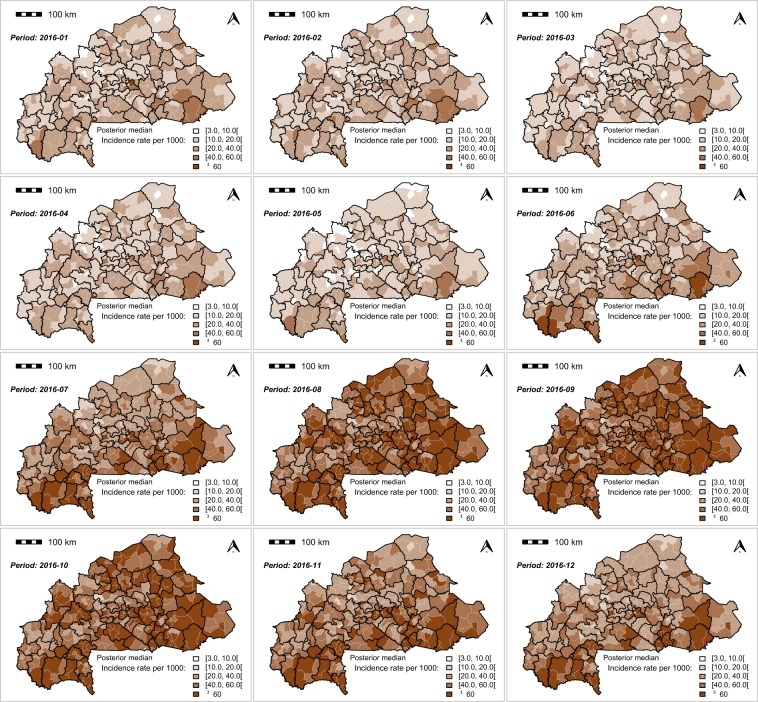
Spatio-temporal dynamic of malaria in pregnancy (MiP) monthly incidence as a rate (per 1000) of fitted values (based on posterior medians) and total number of pregnant women at risk per community for Year 2016. Source: The shapefile was obtained from the “Base Nationale de Découpage du territoire” of Burkina Faso (BNDT, 2006). The malaria in pregnancy data were obtained from the National Malaria Control Program of Burkina Faso, downloaded from the District Health Information System 2. Maps created by Toussaint Rouamba *et al*., 2019.

**Figure 6:**
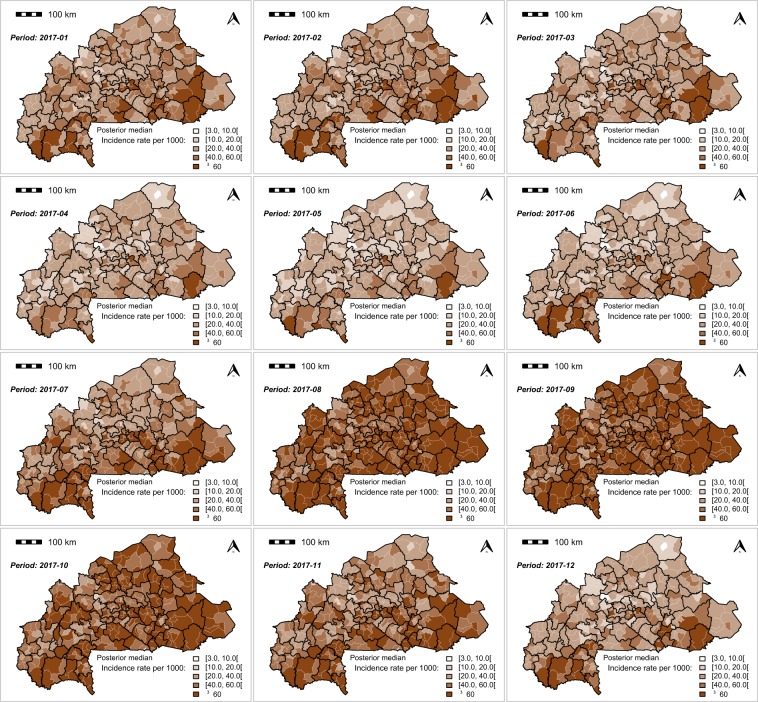
Spatio-temporal dynamic of malaria in pregnancy (MiP) monthly incidence as a rate (per 1000) of fitted values (based on posterior medians) and total number of pregnant women at risk per community for Year 2017. Source: The shapefile was obtained from the “Base Nationale de Découpage du territoire” of Burkina Faso (BNDT, 2006). The malaria in pregnancy data were obtained from the National Malaria Control Program of Burkina Faso, downloaded from the District Health Information System 2. Maps created by Toussaint Rouamba *et al*., 2019.

The monthly MiP posterior incidence rate exhibited a spatial heterogenicity across the country (Figs. 4,5 and 6). The highest MiP posterior incidence rates mainly occurred in the communities located in the east, central-east, Cascades, and south-west regions, whereas the lowest MiP posterior incidence rates mainly occurred in the communities located in the Sahel, north, and central regions.

**Figure 7:**
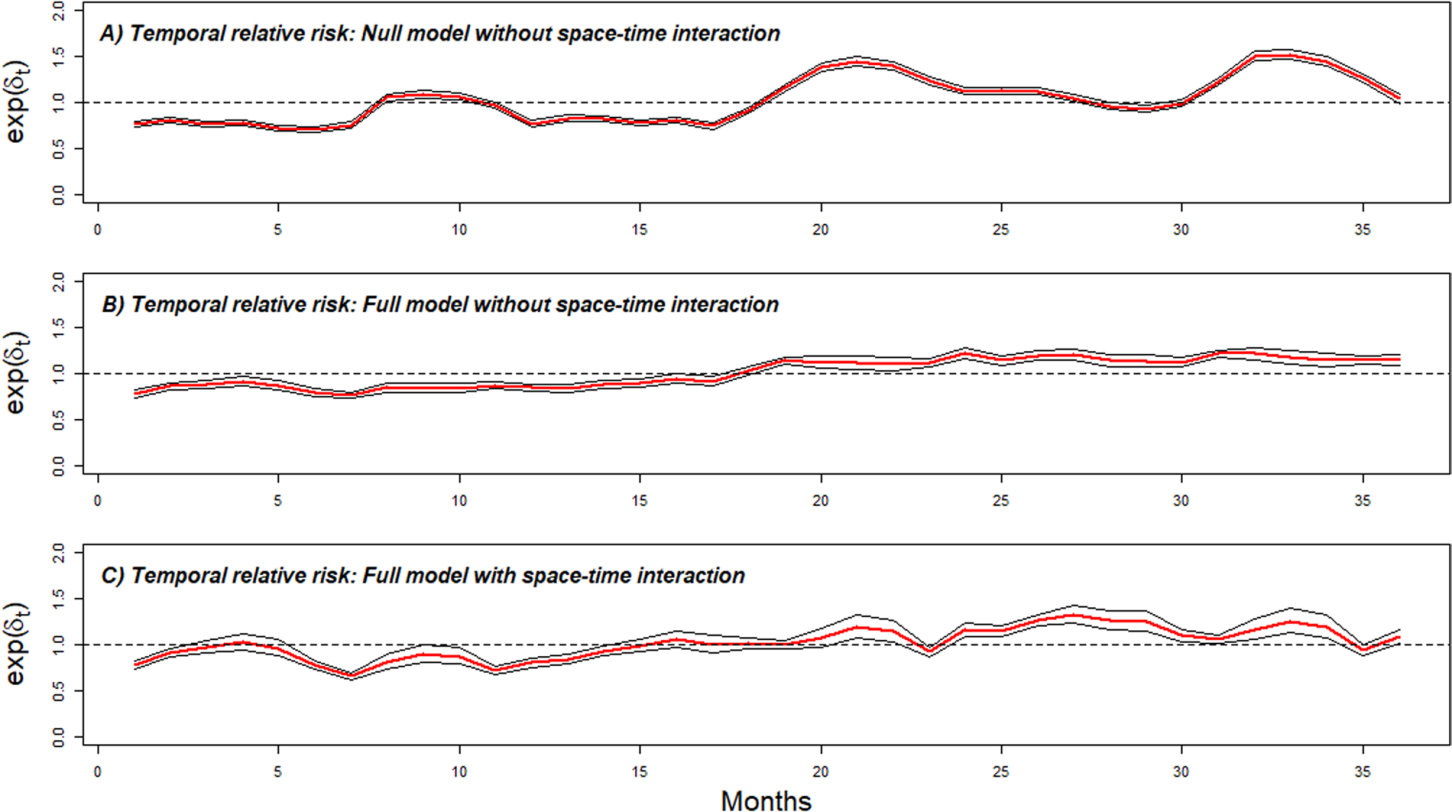
Overall temporal trend (month specific risk) common to all communities exp(δ_t_). Posterior median (red) and 95% credible interval (black). (**A**) Overall temporal trend from null model. (**B**) Overall temporal trend from full model without space-time. (**C**) Overall temporal trend adjusted for covariates effect with space-time. The dashed horizontal black line represents the monthly average of national temporal random effect (January 2015-December 2017).

Figure 7 shows the posterior medians of the average temporal trend of the monthly specific risk (*δ*_*t*_) for the all communities in the MiP incidence rate. The posterior overall temporal trend from the null model fluctuated monthly. Between June 2016 and December 2017 (Fig. 7A–C), the monthly specific risk increased overall. Figure 7c however adjusts for seasonal, covariates and spatial-temporal interactions. The time-series decompositions over the study period of the predicted posterior median incidence rate and case counts are presented in the Fig. S7.

**Figure 8:**
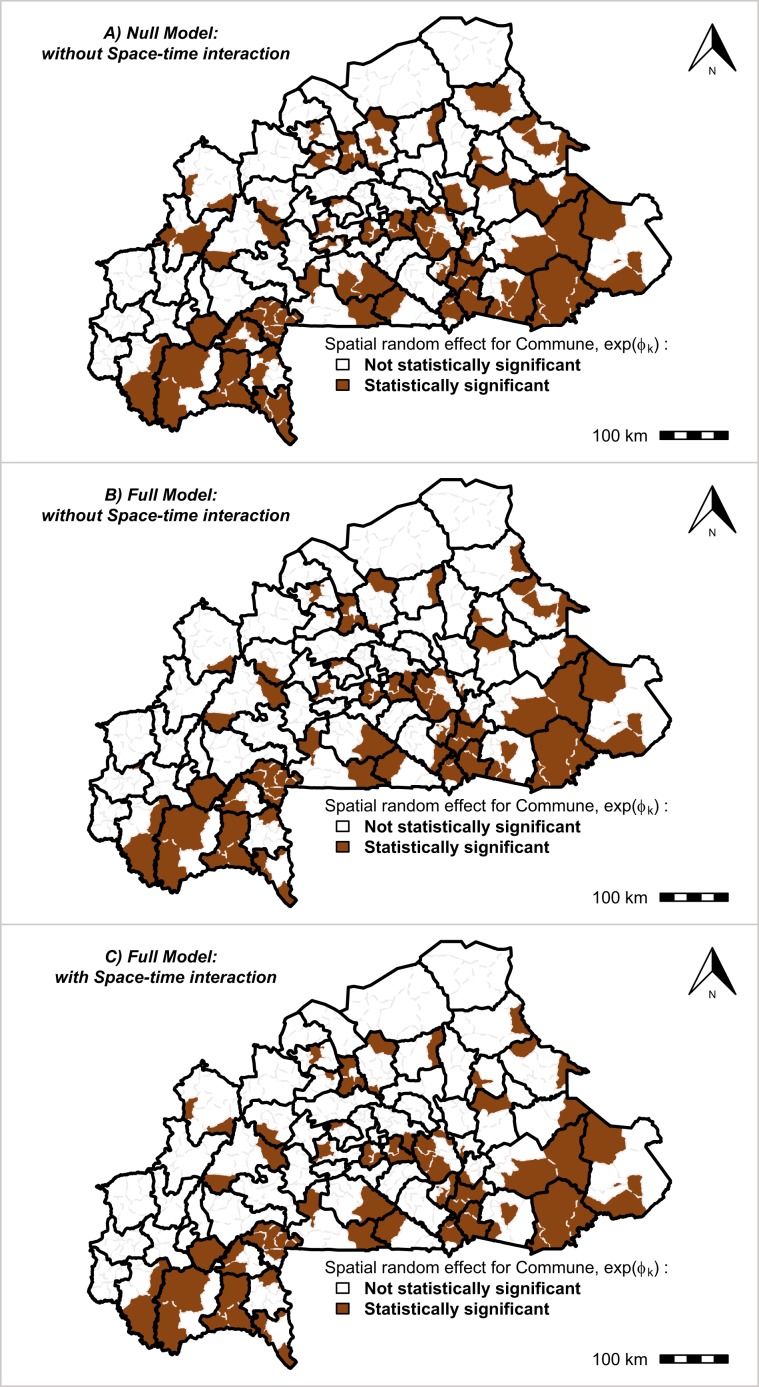
Community specific relative risk of malaria in pregnancy. Overall spatial random effect ${\phi }_{k}$ common to all time periods in each community. Black bold lines correspond to the limits of the health-districts (70 health-districts) and gray lines in each health-district correspond to the limits of communities (351 communities) Source: The shapefile was obtained from the “Base Nationale de Découpage du territoire” of Burkina Faso (BNDT, 2006). The malaria in pregnancy data were obtained from the National Malaria Control Program of Burkina Faso, downloaded from the District Health Information System 2. Maps created by Toussaint Rouamba et al., 2019.

The community spatial risk of MiP was shown to be heterogeneous throughout the country. Figure 8 displays the specific risks of each communities compared with the national mean relative risk (RR = 1). According to the community-specific risks common to all time periods (from 2015 to 2017), the three models indicated some communities where the spatial relative risk was statistically significant, which were mainly located in the Cascades, south-west, center-west, center-east, and east regions. In total, 27.3% (96/351) and 22.5% (79/351) of the communities had spatial relative risk levels that were statistically significant for the model without covariates and the model with covariates and the space–time interaction, respectively (Table S2). Which would indicate that the effect of health programs had reduced the malaria risk in about 17 communities (17.7%). The uncertainty of the monthly case count number of MiP for each community is summarized in the Table S3. The dynamics of the monthly space–time interactions $({Y}_{kt})$ of MiP in each community from 2015 to 2017 are presented in Fig. S8.

### Effects of covariates on incidence rate of MiP

Table 1 summarizes the models evaluating the effects of covariates as well as spatial, temporal, and space–time random effects on the national MiP incidence rate (average of the effect of 351 communities). In models 2 and 3, neither IPTp-SP nor the poverty index had significant effects on the average national MiP incidence rate throughout the study period. Regarding meteorological variables, the results showed that the posterior median and 95% credible interval for the relative risk of a 1 mm increase in rainfall was 1.016 (1.013, 1.019), indicating that an increase of this magnitude corresponds to an increase in the MiP incidence rate of 1.6%, whereas a 1 °C increase in temperature has no significant effect on the MiP incidence rate. The presence of RBF and health promotion projects in a community were associated with increasing the MiP incidence rate, but these effects were not statistically significant. However, the results of the model with covariates (without space–time interaction) showed that communities classified as urban had an additional 10.8% increase in the MiP incidence rate, but this significant increase disappeared after considering the space–time interaction.

## Discussion

This study aimed to quantify morbidity of malaria in pregnant women in Burkina Faso between 2015 and 2017. Our results suggest malaria in pregnancy in Burkina Faso is spatially and temporally heterogeneous. Our modelling was adjusted for the effect of covariates and space-time interaction. Communities located mainly in the Cascades, south-west, central-west, east, and central-east regions exhibited the highest risk levels, whereas communities located in Sahel, Boucle de Mouhoun, and Hauts-bassins exhibited the lowest risk levels. On the one hand, at a national level, health-programs assessed in our study namely IPTp-SP, RBF and Health promotion project did not suggest a change on MiP risk. On the other hand, at local level, some communities have experienced a reduction in their risk resulting from the deployment of these health programs. These findings could help in optimizing interventions in the communities that target pregnant women attending antenatal clinic particularly on testing for malaria early, treatment and tracking infections. In line with national guidelines on IPTp, these findings suggest that prevention measures should be maintained and strengthened during the entire course of pregnancy at any time of the year through social sensitization, and prevention, and surveillance^11,35^.

The increase incidence in malaria amongst pregnant women perhaps correlate with a general increase of incidence in malaria among all population as indicated in the 2019 WHO report^2^. Similar observations were also shown in the annual national health statistics published by the Directorate for Health Statistics of the Ministry of Health, where the annual malaria incidence in the general population (including children under five and pregnant women) increased from 450 to 607 per 1000 people nationally between 2015 and 2017. It is likely that an improvement in data reporting within DHIS 2 correspondent with an apparent increase in number of malaria cases recorded. It is not clear, in terms of magnitude, if access to health facilities in general improved over the study period (2015–2017) due to change in policy by introducing free care for pregnant women in 2016. Thus, a proportional increase in cases recorded during antenatal care visits could be contributed by combination of these factors as well as increased exposure not explored further in this study. As a result, the increase in the number of MiP cases would also suggest underreporting pre-2015^5^.

The high transmission period and geographic distribution of MiP are influenced by the duration of the rainy season, which varies across the country. In the north, the rainy season is short (up to three months); in the central zone, it lasts up to six months; and in the south, it can last up to nine months. Areas with generally high malaria burden^20,22,36^, would also exhibit high MiP. In Burkina Faso, there is limited information on micro-variation of MiP burden at community-level i.e. nationwide studies using the community-level as an observation unit. Since pregnant women represent a vulnerable and an easily accessible population that is being targeted by the NMP, to whom several health programs have been applied, this study provides an overview of the spatio-temporal changes in MiP, and could be used as a proxy for the surveillance of the malaria burden at the community level^7,8,10^.

Bayesian Spatio-temporal modelling showed a spatial dependency (*ρS* = 0.573) and temporal autocorrelation (*ρT* = 0.868). These findings mean that the intensity of the MiP burden from neighborhood communities tends be more similar than that in communities that are further apart^37^. This situation could have some implications for the management and surveillance at a district-level and can be replicated in the population of children aged less than five or in the general population. Notably, spatial and temporal (with lag times) dependency must be considered as information sources rather than something to be corrected^38^; knowing this, the local authorities of neighboring communities, in tandem with local health managers, should make concerted efforts to improve routine health information systems (systematic collection of data, analysis, and interpretation) to improve and strengthen existing measures for the achievement of the Sustainable Development Goal 3 (SDG3). In addition, the global stability of the temporal specific risk highlights the need to maintain prevention and treatment measures and increase women’s sensitization throughout the year. Finally, the NMP should integrate the geographic information system into routine surveillance programs to regularly produce and update maps of malaria risk generally, and malaria burden during pregnancy particularly.

In Burkina Faso, we found that the MiP peaked between August and November (Fig. S7) and is positively and significantly correlated at lagged months 1, 2, and 3 of rainfalls. Likewise, spatial modeling showed that rainfall is significantly associated with an increase in the MiP incidence rate. These findings could contribute to better understanding the role and the suitable lag-times of meteorological factors on the spatio-temporal distribution of MiP throughout the country. These lag-times highlighted that, generally, following the first rainfall and due to infiltration or evaporation, the creation of a temporary water body requires numerous episodes of rain to reach the level required for *Anopheles* breeding sites^39,40^. The seasonal lag-times highlighted in our study could be relevant for the NMP development of malaria vector interventions, such as deploying indoor residual spraying (IRS) during months of high incidence of each year. In addition, in Burkina Faso context and especially in rural area, the beginning of “farming hamlets” and activities related to agriculture follows the seasonally of rains and people including women (pregnant or not) move to these agricultural hamlets for performing agricultural activities and return to their usual homesteads during the dry season. The location of these hamlets, usually far from health facilities, would hinder the accessibility of pregnant women to malaria prevention measures (especially IPTp-SP) during ANCs^41^. At the same time, the poor housing conditions in these temporary farming hamlets would not be suitable for reduction of vector densities (entry of malaria vectors indoors) and entomological inoculation rates^42,43^.

The combined effect (in term of significant reducing effect) of RBF program, health promotion projects and IPTp-SP strategies was greatest in high burden malaria communities (Table S2 and Fig. 8). For example, in 17 (17.7%, 17/96) communities (Sami, Solenzo, Sangha, Soudougui, Koupéla, Kongoussi, Boulsa, Sabou, Cassou, Gayéri, Gourcy, Dori, Sebba, Diebougou, Zambo, Batié, and Gaoua) where the community-specific risk was statistically significant. These findings suggest that targeted response as advocated for high burden (HBHI) countries could be beneficial^44,45^.

One of the strengths of our study is the type of analysis used to explore the spatio-temporal variations in the MiP burden through routinely collected observational health data. As recommended in pillar 3 of the WHO’s global technical strategy (GTS) for malaria^46^, Burkina Faso, being a high burden country, requires routine surveillance and analysis of monthly-aggregated data to transform existing data into crucial information that can be used by the NMP to direct resources toward the most affected areas and populations and to evaluate the effectiveness of health programs^47^. The application of the hierarchical Bayesian CAR approach with data from several sources can address unknown sources of bias. Likewise, the CAR model smooths the MiP incidence, considering the potential impact of model instability caused by the small number of reported cases and the diversity of data sources^48,49^.

Although this analysis achieved our aims by providing robust estimates, it is important to stress that malaria infection among pregnant women living in areas of high malaria transmission is often asymptomatic, so malaria may be difficult to recognize and diagnose^50^. Indeed, MiP is characterized by the sequestration of parasites in the placenta, which are often undetectable in peripheral blood smears using classical methods (RDT or microscopy), even under optimal conditions^51^. Thus, the number of MiP cases reported in the DHIS 2 could be a portion of the actual cases and would represent only a fraction of the total^52^. However, in Burkina Faso, where the rate of ANC coverage is high, since pregnant women regularly attend ANC, more opportunities exist to detect cases. This situation provides an opportunity for the NMP to use pregnant women as a sentinel population for malaria surveillance as proposed elsewhere^8–10^. In addition, the numbers of pregnancies at risk in each community for the years 2016 and 2017 were projected from national census data from 2006^27^, and this may have led to over- or under-estimation of the number of pregnancies at risk. The two broad interventions were implemented in only one year and at the beginning of the study period: in 2014 and 2015 for RBF and the health promotion project, respectively. These intervention variables are binary variables, so a certain amount of time would be required for these interventions to yield their full effects. Our findings should be considered with these strengths and limitations in mind.

## Conclusion

Despite the intensification of malaria control efforts, MiP cases remain high at the community level with a heterogeneous risk across the country. Burkina Faso’s national malaria strategy aims to reduce malaria cases and deaths by at least 40% by 2020 from the 2015 level^53^. This study suggests a stalling of the reduction of malaria among pregnant women between 2015 and 2017 and national goals may not be achieved. The NMP of Burkina Faso including other endemic countries, in their effort to optimize malaria burden surveillance and assess the progress of health programs should integrate a disease modeling approach and a geographic information system in a routine surveillance platform to promote the production and regular update of information on the malaria risk and the MiP burden. This disease modelling for monitoring purposes should be accompanied by strengthening existing control strategies nationwide, especially community-health promotion activities. This integrated approach (routine data collection such as malaria data and meteorological data, health program data and disease modelling) would help to 1) refine and target resource allocation according to the risk of areas and 2) provide health information in real time.

## Supplementary information

Supplementary Table S1

Supplementary Table S2

Supplementary information3

Supplementary information4

Supplementary information5

Supplementary information6

Supplementary information7

Supplementary information8

Supplementary Table S3

## Data Availability

The dataset regarding malaria in pregnancy cases and Intermittent-treatment-prevention with Sulphadoxine Pyrimethamine are downloaded from at the DHIS2 (https://burkina.dhis2.org) managed by the Directorate General of Studies and Sectoral Statistics of the Burkina Faso Ministry of Health. These later datasets used and/or analyzed during the current study are available from the corresponding author on reasonable request and approbation of Directorate General of Studies and Sectoral Statistics of the Ministry of Health.
