# Supplementary material for: Bayesian Spatiotemporal Modeling of Routinely Collected Data to Assess the Effect of Health Programs in Malaria Incidence During Pregnancy in Burkina Faso
Source: Sci Rep. 2020 Feb 14;10:2618. doi: 10.1038/s41598-020-58899-3 (PMC7021681; doi:10.1038/s41598-020-58899-3)
Supplement: Supplementary file 1 — Supplementary Table S1 [file 41598_2020_58899_MOESM1_ESM.pdf]

**Bayesian Spatiotemporal Modeling of Routinely Collected Data to Assess the Effect of Health Programs in Malaria Incidence During Pregnancy in Burkina Faso**

**Toussaint Rouamba[1,2], Sekou Samadoulougou[3,4], Halidou Tinto[1], Victor A. Alegana[5,6], and Fati Kirakoya–Samadoulougou[2]**

1 Clinical Research Unit of Nanoro, Institute for Research in Health Sciences, National Center for Scientific and Technological Research, 528, Avenue Kumda-Yoore, BP 218 Ouagadougou CMS 11, Ouagadougou, Burkina Faso
2 Center for research in epidemiology, Biostatistics and Clinical Research, School of Public Health, University libre de Bruxelles (ULB), Route de Lennik, 808 B-1070 Bruxelles. Brussels, Belgium
3 Centre for Research on Planning and Development (CRAD), Laval University, Quebec, G1V 0A6, Canada
4 Evaluation Platform on Obesity Prevention, Quebec Heart and Lung Institute, Quebec, G1V 4G5, Canada.
5.Kenya Medical Research Institute - Wellcome Trust Research Programme, P.O. Box 43640-00100, Nairobi, Kenya
6. Geography and Environmental Science, University of Southampton, SO17 1BJ Southampton, UK

*Correspondence to rouambatoussaint@gmail.com

**Supplementary Information 1**

**Table S1.** Monthly Moran's *I* statistics values obtained from the residuals of a non-spatial multivariable over-dispersed Poisson log-linear model

| | 2015 | | 2016 | | 2017 | |
|---|---|---|---|---|---|---|
| | Statistic | P value | Statistic | P value | Statistic | P value |
| January | 0.016 | 0.25 | 0.132 | **<0.001** | 0.055 | **0.045** |
| February | 0.060 | **0.028** | -0.017 | 0.89 | 0.0749 | **0.013** |
| March | 0.166 | **<0.001** | 0.019 | 0.20 | 0.081 | **0.006** |
| April | 0.038 | 0.09 | 0.156 | **<0.001** | 0.096 | **0.003** |
| May | 0.044 | 0.07 | 0.125 | **<0.001** | 0.061 | **0.030** |
| June | -0.018 | 0.67 | 0.033 | 0.11 | 0.121 | **<0.001** |
| July | 0.028 | 0.10 | 0.062 | **0.029** | 0.165 | **<0.001** |
| August | 0.090 | **0.007** | 0.028 | 0.16 | 0.157 | **<0.001** |
| September | 0.057 | **0.04** | 0.077 | **0.013** | 0.233 | **<0.001** |
| October | 0.055 | **0.040** | 0.086 | **0.008** | 0.115 | **0.002** |
| November | 0.063 | **0.035** | 0.111 | **0.002** | 0.122 | **<0.001** |
| December | -0.011 | 0.58 | 0.077 | **0.013** | 0.144 | **<0.001** |
