# Supplementary material for: Bayesian Spatiotemporal Modeling of Routinely Collected Data to Assess the Effect of Health Programs in Malaria Incidence During Pregnancy in Burkina Faso
Source: Sci Rep. 2020 Feb 14;10:2618. doi: 10.1038/s41598-020-58899-3 (PMC7021681; doi:10.1038/s41598-020-58899-3)
Supplement: Supplementary file 2 — Supplementary Table S2 [file 41598_2020_58899_MOESM2_ESM.pdf]

Bayesian Spatiotemporal Modeling of Routinely Collected Data to Assess the Effect of Health Programs in Malaria Incidence During Pregnancy in Burkina Faso

Toussaint Rouamba[1,2], Sekou Samadoulougou[3,4], Halidou Tinto[1], Victor A. Alegana[5,6], and Fati Kirakoya–Samadoulougou[2]

1 Clinical Research Unit of Nanoro, Institute for Research in Health Sciences, National Center for Scientific and Technological Research, 528, Avenue Kumda-Yoore, BP 218 Ouagadougou CMS 11, Ouagadougou, Burkina Faso
2 Center for research in epidemiology, Biostatistics and Clinical Research, School of Public Health, University libre de Bruxelles (ULB), Route de Lennik, 808 B-1070 Bruxelles. Brussels, Belgium
3 Centre for Research on Planning and Development (CRAD), Laval University, Quebec, G1V 0A6, Canada
4 Evaluation Platform on Obesity Prevention, Quebec Heart and Lung Institute, Quebec, G1V 4G5, Canada.
5.Kenya Medical Research Institute - Wellcome Trust Research Programme, P.O. Box 43640-00100, Nairobi, Kenya
6. Geography and Environmental Science, University of Southampton, SO17 1BJ Southampton, UK

*Correspondence to rouambatoussaint@gmail.com

Supplementary Information 2

Table S2. Malaria in pregnancy high burden Community in Burkina Faso between 2015 – 2017: Communities specific risks related to National risk computed from Model without covariates and Model with covariates and space-time interaction.

Region	Commune	Model without covariates	Model with covariates and space-time interaction
		RR (95% CrI)	RR (95% CrI)
Boucle du Mouhoun	Sami	1.16 (1.01, 1.31)	
	Solenzo	1.16 (1.08, 1.25)	
	Bourasso	1.21 (1.07, 1.37)	1.20 (1.05, 1.38)
	Doumbala	1.14 (1.03, 1.27)	1.14 (1.01, 1.28)
	Bondokuy	1.18 (1.08, 1.28)	1.20 (1.09, 1.31)
	Tchériba	1.14 (1.04, 1.25)	1.18 (1.07, 1.30)
	Ye	1.13 (1.02, 1.24)	1.17 (1.05, 1.29)
Cascades	Mangodara	1.36 (1.22, 1.51)	1.30 (1.16, 1.43)
	Niangoloko	1.48 (1.35, 1.62)	1.31 (1.19, 1.44)
	Sidéradougou	1.38 (1.27, 1.50)	1.35 (1.23, 1.48)
	Tiéfora	1.46 (1.34, 1.59)	1.44 (1.31, 1.58)
Center–East	Bagre	1.49 (1.36, 1.63)	1.46 (1.31, 1.62)
	Bane	1.42 (1.28, 1.57)	1.44 (1.30, 1.61)
	Beguedo	1.19 (1.06, 1.34)	1.21 (1.07, 1.38)
	Bittou	1.27 (1.17, 1.38)	1.16 (1.05, 1.29)
	Boussouma	1.27 (1.15, 1.41)	1.30 (1.17, 1.44)

Region	Commune	Model without covariates	Model with covariates and space-time interaction
		RR (95% CrI)	RR (95% CrI)
	Garango	1.19 (1.10, 1.29)	1.11 (1.01, 1.22)
	Tenkodogo	1.46 (1.35, 1.59)	1.31 (1.19, 1.44)
	Zabré	1.35 (1.23, 1.48)	1.37 (1.24, 1.51)
	Zonsé	1.37 (1.23, 1.53)	1.40 (1.24, 1.56)
	Sangha	1.15 (1.04, 1.27)	
	Soudougui	1.13 (1.03, 1.24)	
	Yargatenga	1.40 (1.27, 1.56)	1.35 (1.20, 1.51)
	Yondé	1.22 (1.10, 1.35)	1.18 (1.05, 1.33)
	Dialgaye	1.26 (1.13, 1.39)	1.28 (1.15, 1.43)
	Koupéla	1.16 (1.06, 1.26)	
	Pouytenga	1.26 (1.14, 1.39)	1.23 (1.09, 1.39)
Center−North	Bourzanga	1.23 (1.12, 1.34)	1.23 (1.11, 1.36)
	Guibare	1.20 (1.08, 1.34)	1.20 (1.06, 1.34)
	Kongoussi	1.10 (1.01, 1.20)	
	Boulsa	1.15 (1.06, 1.25)	
	Pensa	1.19 (1.07, 1.32)	1.26 (1.12, 1.40)
	Koudougou	1.44 (1.32, 1.57)	1.33 (1.18, 1.49)
	Nanoro	1.21 (1.09, 1.34)	1.25 (1.08, 1.44)
	Sabou	1.16 (1.05, 1.27)	
	Bieha	1.30 (1.17, 1.43)	1.26 (1.12, 1.41)
	Silly	1.15 (1.04, 1.27)	1.13 (1.01, 1.27)
	Cassou	1.13 (1.02, 1.24)	
	Sapouy	1.30 (1.19, 1.41)	1.14 (1.02, 1.27)
	Guiaro	1.20 (1.06, 1.35)	1.21 (1.07, 1.36)
	Komki-Ipala	1.27 (1.14, 1.42)	1.30 (1.15, 1.46)
	Komsilga	1.44 (1.32, 1.57)	1.46 (1.28, 1.66)
Center	Koubri	1.40 (1.28, 1.52)	1.43 (1.30, 1.57)
	Saaba	1.73 (1.58, 1.89)	1.75 (1.53, 2.00)
	Tanghin-Dassouri	1.17 (1.07, 1.28)	1.19 (1.07, 1.32)
	Bogandé	1.22 (1.13, 1.32)	1.14 (1.03, 1.26)
	Mani	1.19 (1.09, 1.31)	1.25 (1.14, 1.38)
	Fada N'gourma	1.44 (1.33, 1.56)	1.34 (1.23, 1.46)
	Matiacoali	1.39 (1.28, 1.51)	1.44 (1.31, 1.58)
	Foutouri	1.29 (1.15, 1.45)	1.35 (1.19, 1.53)
	Gayéri	1.14 (1.04, 1.25)	
East	Kompienga	1.50 (1.34, 1.69)	1.52 (1.35, 1.71)
	Madjoari	1.51 (1.33, 1.71)	1.54 (1.35, 1.77)
	Pama	1.62 (1.48, 1.77)	1.49 (1.34, 1.66)
	Kantchari	1.12 (1.02, 1.23)	1.18 (1.06, 1.31)
	Logobou	1.14 (1.04, 1.24)	1.16 (1.04, 1.31)
	Namounou	1.42 (1.25, 1.60)	1.44 (1.27, 1.64)
	Tansarga	1.35 (1.22, 1.49)	1.40 (1.24, 1.58)

Region	Commune	Model without covariates	Model with covariates and space-time interaction
		RR (95% CrI)	RR (95% CrI)
Hauts–Bassins	Karankasso-Vigué	1.19 (1.10, 1.29)	1.19 (1.09, 1.29)
North	Kalsaka	1.11 (1.01, 1.22)	1.16 (1.05, 1.28)
	Ouahigouya	1.28 (1.19, 1.38)	1.21 (1.08, 1.36)
	Rambo	1.14 (1.03, 1.26)	1.20 (1.08, 1.33)
	Seguenega	1.23 (1.13, 1.34)	1.29 (1.18, 1.40)
	Bassi	1.23 (1.10, 1.36)	1.22 (1.08, 1.37)
	Gourcy	1.14 (1.05, 1.23)	
Plateau–Central	Boudry	1.21 (1.11, 1.31)	1.24 (1.14, 1.36)
	Mogtédo	1.17 (1.07, 1.29)	1.21 (1.09, 1.33)
	Salogo	1.13 (1.01, 1.27)	1.17 (1.04, 1.32)
	Zam	1.22 (1.10, 1.34)	1.25 (1.13, 1.39)
	Nagreongo	1.19 (1.06, 1.33)	1.22 (1.09, 1.37)
Sahel	Dori	1.13 (1.04, 1.22)	
	Seytenga		1.15 (1.03, 1.29)
	Boundore	1.17 (1.04, 1.31)	1.23 (1.09, 1.39)
	Sebba	1.22 (1.10, 1.35)	
	Titabe	1.42 (1.28, 1.57)	1.49 (1.34, 1.66)
South–west	Bondigui	1.40 (1.25, 1.56)	1.34 (1.19, 1.50)
	Diebougou	1.15 (1.04, 1.26)	
	Gueguere	1.47 (1.34, 1.61)	1.47 (1.33, 1.62)
	Iolonioro	1.21 (1.08, 1.36)	1.16 (1.03, 1.32)
	Dano	1.38 (1.27, 1.51)	1.26 (1.14, 1.40)
	Dissin	1.20 (1.09, 1.33)	1.20 (1.06, 1.35)
	Koper	1.28 (1.14, 1.43)	1.27 (1.12, 1.44)
	Niego	1.38 (1.20, 1.58)	1.37 (1.19, 1.58)
	Oronkua	1.28 (1.15, 1.43)	1.29 (1.15, 1.45)
	Ouessa	1.91 (1.70, 2.13)	1.90 (1.68, 2.15)
	Zambo	1.16 (1.03, 1.30)	
	Batié	1.14 (1.02, 1.26)	
	Boussoukoula	1.96 (1.72, 2.22)	1.79 (1.55, 2.07)
	Kpuéré	1.72 (1.48, 1.98)	1.55 (1.33, 1.83)
	Legmoin	1.35 (1.19, 1.53)	1.24 (1.08, 1.42)
	Midebdo	1.44 (1.28, 1.62)	1.33 (1.17, 1.52)
	Djigouè	1.43 (1.28, 1.60)	1.39 (1.23, 1.57)
	Gaoua	1.12 (1.02, 1.22)	
	Kampti	1.36 (1.23, 1.50)	1.31 (1.19, 1.45)
	Loropeni	1.35 (1.23, 1.47)	1.33 (1.21, 1.46)
	Nako	1.23 (1.11, 1.37)	1.23 (1.10, 1.39)

RR: Relative risk, 95% CrI: 95% credible intervals

Example, a RR value of 1.23 corresponds to a 23% increased risk of malaria in pregnancy
