# Supplementary material for: Bayesian Spatiotemporal Modeling of Routinely Collected Data to Assess the Effect of Health Programs in Malaria Incidence During Pregnancy in Burkina Faso
Source: Sci Rep. 2020 Feb 14;10:2618. doi: 10.1038/s41598-020-58899-3 (PMC7021681; doi:10.1038/s41598-020-58899-3)
Supplement: Supplementary file 3 — Supplementary information3 [file 41598_2020_58899_MOESM3_ESM.pdf]

Bayesian Spatiotemporal Modeling of Routinely Collected Data to Assess the Effect of Health Programs in Malaria Incidence During Pregnancy in Burkina Faso

Toussaint Rouamba[1,2], Sekou Samadoulougou[3,4], Halidou Tinto[1], Victor A. Alegana[5,6], and Fati Kirakoya–Samadoulougou[2]

1 Clinical Research Unit of Nanoro, Institute for Research in Health Sciences, National Center for Scientific and Technological Research, 528, Avenue Kumda-Yoore, BP 218 Ouagadougou CMS 11, Ouagadougou, Burkina Faso

2 Center for research in epidemiology, Biostatistics and Clinical Research, School of Public Health, University libre de Bruxelles (ULB), Route de Lennik, 808 B-1070 Bruxelles. Brussels, Belgium

3 Centre for Research on Planning and Development (CRAD), Laval University, Quebec, G1V 0A6, Canada

4 Evaluation Platform on Obesity Prevention, Quebec Heart and Lung Institute, Quebec, G1V 4G5, Canada.

5.Kenya Medical Research Institute - Wellcome Trust Research Programme, P.O. Box 43640-00100, Nairobi, Kenya

6. Geography and Environmental Science, University of Southampton, SO17 1BJ Southampton, UK

*Correspondence to rouambatoussaint@gmail.com

Supplementary Information 3

Figure S1. Map showing the geographic distribution of health program in Burkina Faso. A) Results Based Financing for Health was implemented in 2014 and B) Health promotion in 130 communities project" implemented in 2015. Source: The shapefile was obtained from the "Base Nationale de Découpage du territoire" of Burkina Faso (BNDT, 2006). The data were obtained from the Ministry of Health of Burkina Faso. Maps created by Toussaint Rouamba et al, 2019

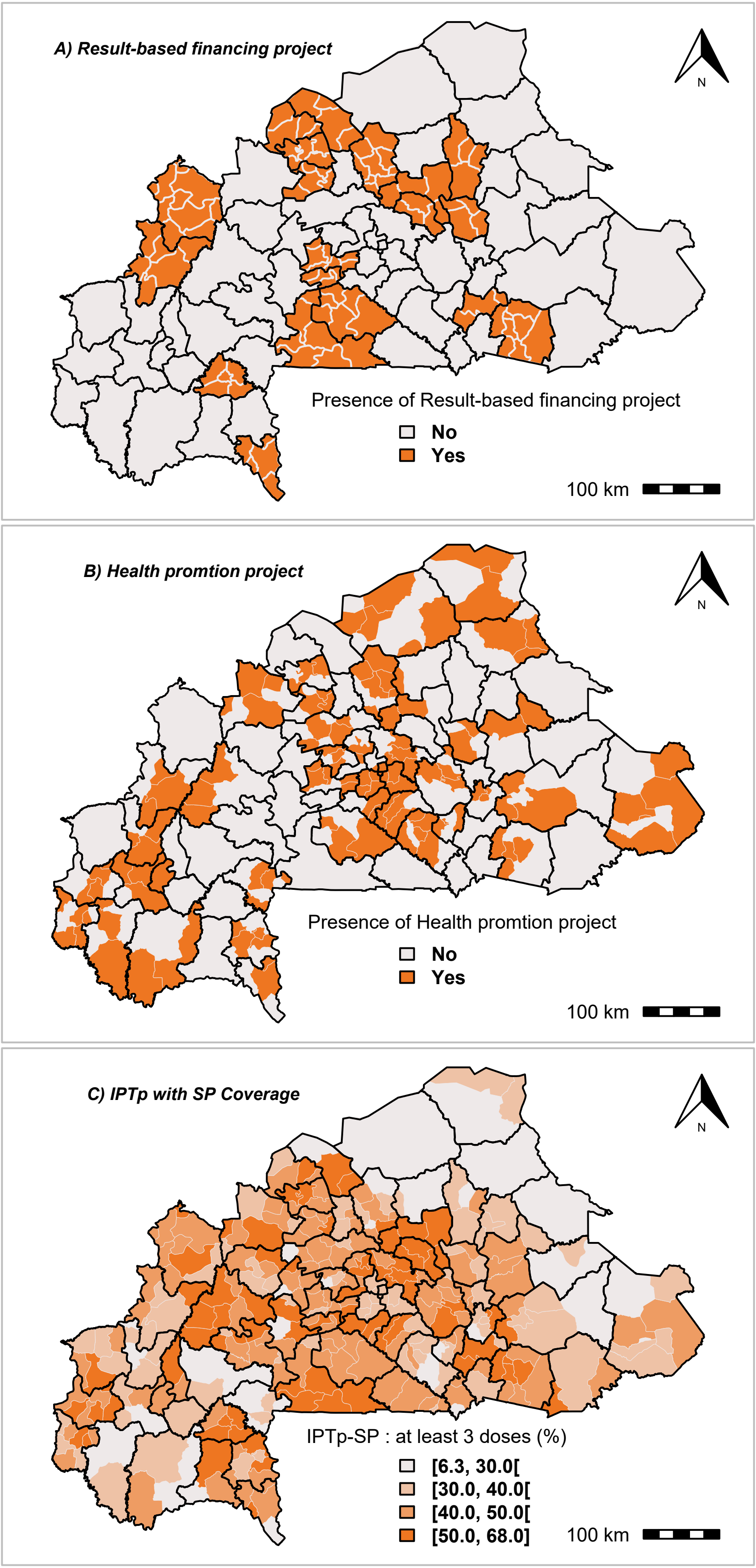

A) Result-based financing project
N
Presence of Result-based financing project
No
Yes
100 km
B) Health promtion project
N
Presence of Health promtion project
No
Yes
100 km
C) IPTp with SP Coverage
N
IPTp-SP : at least 3 doses (%)
[6.3, 30.0[
[30.0, 40.0[
[40.0, 50.0[
[50.0, 68.0]
100 km
