# Supplementary material for: Bayesian Spatiotemporal Modeling of Routinely Collected Data to Assess the Effect of Health Programs in Malaria Incidence During Pregnancy in Burkina Faso
Source: Sci Rep. 2020 Feb 14;10:2618. doi: 10.1038/s41598-020-58899-3 (PMC7021681; doi:10.1038/s41598-020-58899-3)
Supplement: Supplementary file 6 — Supplementary information6 [file 41598_2020_58899_MOESM6_ESM.pdf]

Bayesian Spatiotemporal Modeling of Routinely Collected Data to Assess the Effect of Health Programs in Malaria Incidence During Pregnancy in Burkina Faso

Toussaint Rouamba[1,2], Sekou Samadoulougou[3,4], Halidou Tinto[1], Victor A. Alegana[5,6], and Fati Kirakoya–Samadoulougou[2]

1 Clinical Research Unit of Nanoro, Institute for Research in Health Sciences, National Center for Scientific and Technological Research, 528, Avenue Kumda-Yoore, BP 218 Ouagadougou CMS 11, Ouagadougou, Burkina Faso
2 Center for research in epidemiology, Biostatistics and Clinical Research, School of Public Health, University libre de Bruxelles (ULB), Route de Lennik, 808 B-1070 Bruxelles. Brussels, Belgium
3 Centre for Research on Planning and Development (CRAD), Laval University, Quebec, G1V 0A6, Canada
4 Evaluation Platform on Obesity Prevention, Quebec Heart and Lung Institute, Quebec, G1V 4G5, Canada.
5.Kenya Medical Research Institute - Wellcome Trust Research Programme, P.O. Box 43640-00100, Nairobi, Kenya
6. Geography and Environmental Science, University of Southampton, SO17 1BJ Southampton, UK

*Correspondence to rouambatoussaint@gmail.com

Supplementary Information 6

Figure S6. Posterior distributions of the regression parameters on antilog scale. The left panel contains trace-plots while the right panel are density estimates.

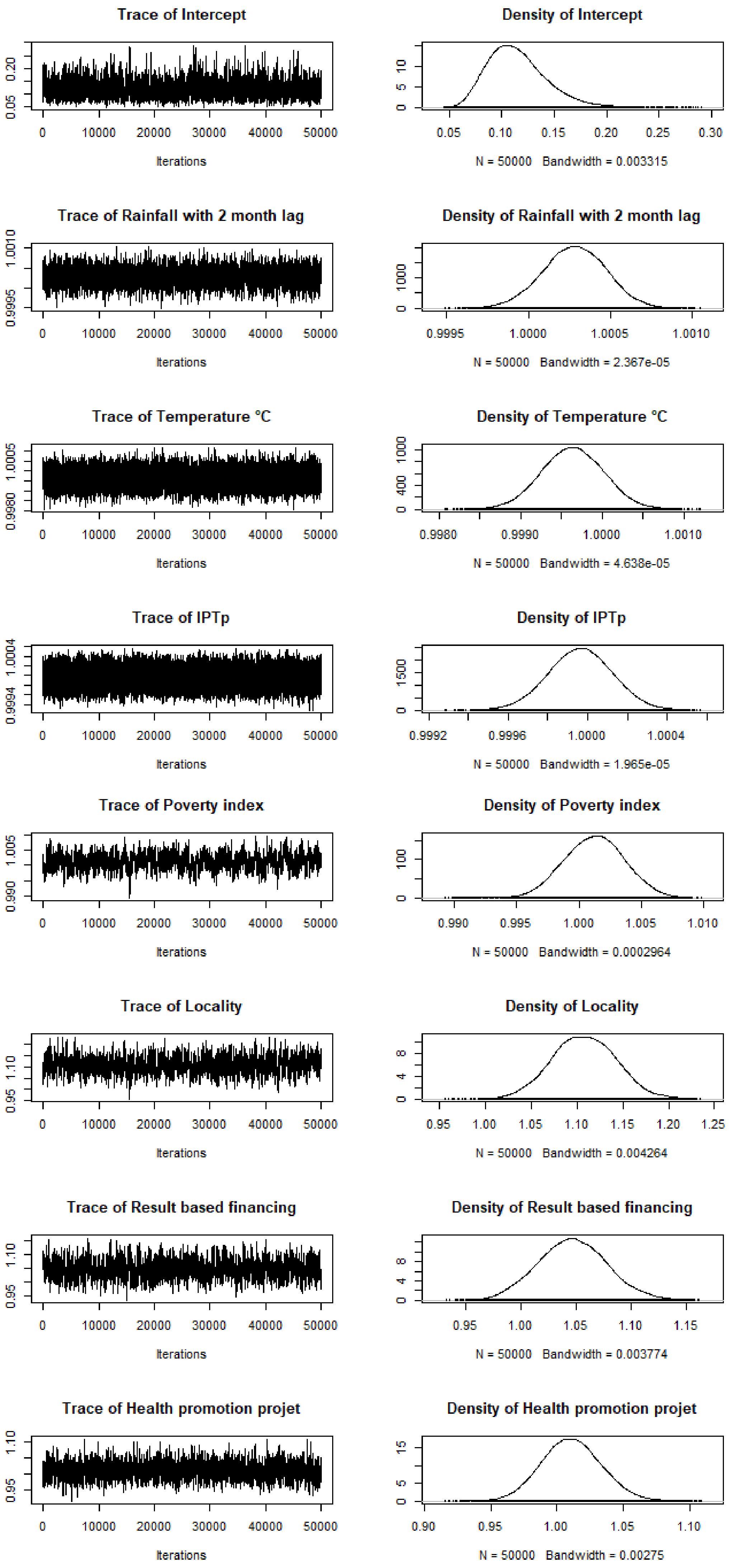
Trace of Intercept
0.05
0.20
0
10000
20000
30000
40000
50000
Iterations
Density of Intercept
0
5
10
0.05
0.10
0.15
0.20
0.25
0.30
N = 50000 Bandwidth = 0.003315
Trace of Rainfall with 2 month lag
0.9995
1.0010
Iterations
Density of Rainfall with 2 month lag
0
1000
0.9995
1.0000
1.0005
1.0010
N = 50000 Bandwidth = 2.367e-05
Trace of Temperature °C
0.9980
1.0005
Iterations
Density of Temperature °C
0
400
1000
0.9980
0.9990
1.0000
1.0010
N = 50000 Bandwidth = 4.638e-05
Trace of IPTp
0.9994
1.0004
Iterations
Density of IPTp
0
1500
0.9992
0.9996
1.0000
1.0004
N = 50000 Bandwidth = 1.965e-05
Trace of Poverty index
0.990
1.005
Iterations
Density of Poverty index
0
100
0.990
0.995
1.000
1.005
1.010
N = 50000 Bandwidth = 0.0002964
Trace of Locality
0.95
1.10
Iterations
Density of Locality
0
4
8
0.95
1.00
1.05
1.10
1.15
1.20
1.25
N = 50000 Bandwidth = 0.004264
Trace of Result based financing
0.95
1.10
Iterations
Density of Result based financing
0
4
8
0.95
1.00
1.05
1.10
1.15
N = 50000 Bandwidth = 0.003774
Trace of Health promotion projet
0.95
1.10
Iterations
Density of Health promotion projet
0
5
15
0.90
0.95
1.00
1.05
1.10
N = 50000 Bandwidth = 0.00275
