# Supplementary material for: Bayesian Spatiotemporal Modeling of Routinely Collected Data to Assess the Effect of Health Programs in Malaria Incidence During Pregnancy in Burkina Faso
Source: Sci Rep. 2020 Feb 14;10:2618. doi: 10.1038/s41598-020-58899-3 (PMC7021681; doi:10.1038/s41598-020-58899-3)
Supplement: Supplementary file 7 — Supplementary information7 [file 41598_2020_58899_MOESM7_ESM.pdf]

Bayesian Spatiotemporal Modeling of Routinely Collected Data to Assess the Effect of Health Programs in Malaria Incidence During Pregnancy in Burkina Faso

Toussaint Rouamba[1,2], Sekou Samadoulougou[3,4], Halidou Tinto[1], Victor A. Alegana[5,6], and Fati Kirakoya–Samadoulougou[2]

1 Clinical Research Unit of Nanoro, Institute for Research in Health Sciences, National Center for Scientific and Technological Research, 528, Avenue Kumda-Yoore, BP 218 Ouagadougou CMS 11, Ouagadougou, Burkina Faso
2 Center for research in epidemiology, Biostatistics and Clinical Research, School of Public Health, University libre de Bruxelles (ULB), Route de Lennik, 808 B-1070 Bruxelles. Brussels, Belgium
3 Centre for Research on Planning and Development (CRAD), Laval University, Quebec, G1V 0A6, Canada
4 Evaluation Platform on Obesity Prevention, Quebec Heart and Lung Institute, Quebec, G1V 4G5, Canada.
5.Kenya Medical Research Institute - Wellcome Trust Research Programme, P.O. Box 43640-00100, Nairobi, Kenya
6. Geography and Environmental Science, University of Southampton, SO17 1BJ Southampton, UK

*Correspondence to rouambatoussaint@gmail.com

Supplementary Information 7

Figure S7. Malaria in pregnancy monthly posterior median incidence per 1000 and posterior median case counts per month during the study period (2015–2017). The top layer shows the temporal pattern in posterior median time series (reported cases). The bottom left layer shows the decomposed components into posterior median seasonal and trend component. The bottom right layer shows decomposed remainder component.

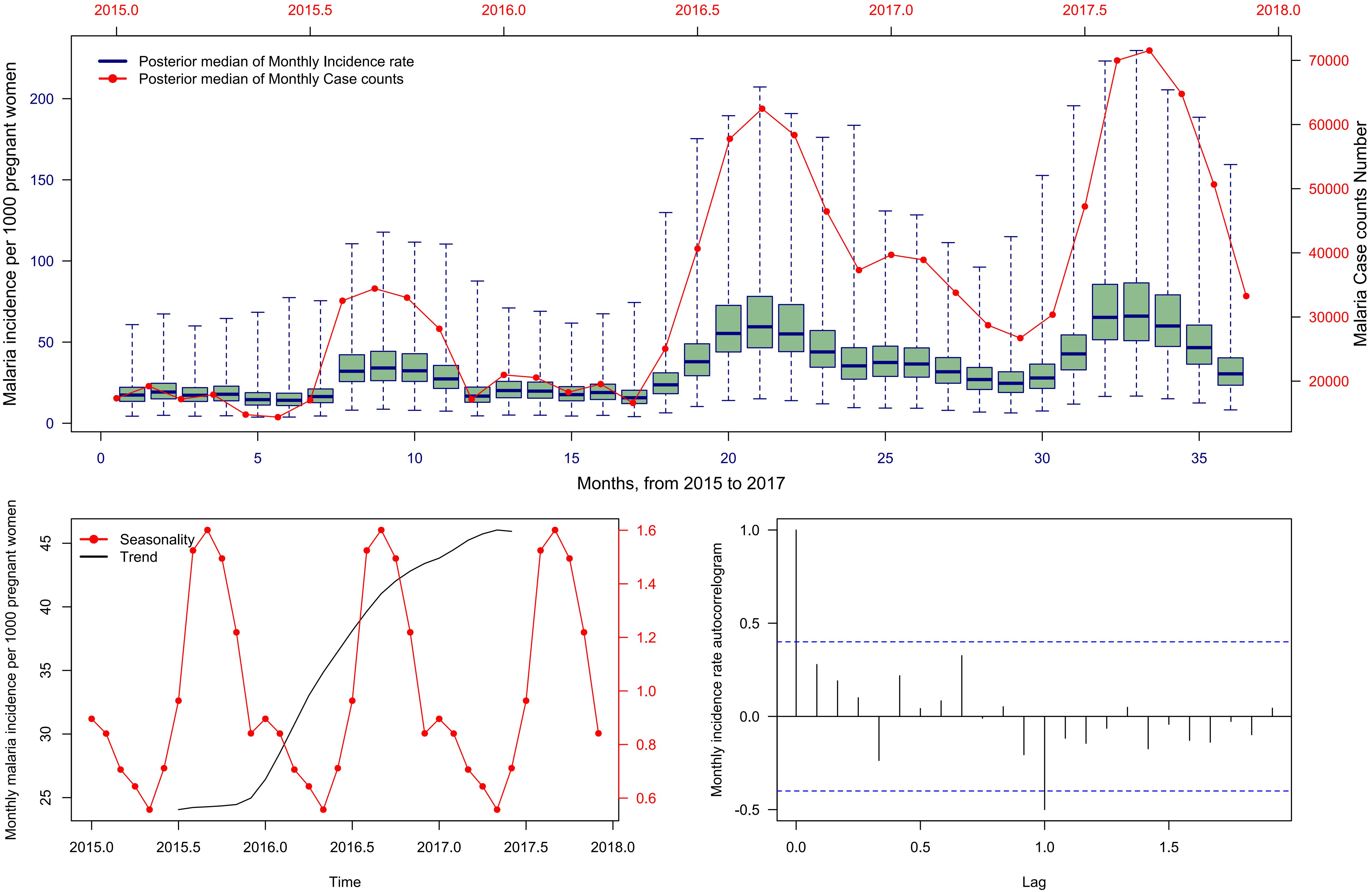

Posterior median of Monthly Incidence rate
Posterior median of Monthly Case counts
Malaria incidence per 1000 pregnant women
Malaria Case counts Number
Months, from 2015 to 2017
2015.0
2015.5
2016.0
2016.5
2017.0
2017.5
2018.0
Seasonality
Trend
Monthly malaria incidence per 1000 pregnant women
Time
Monthly incidence rate autocorrelogram
Lag
