# Supplementary material for: Bayesian Spatiotemporal Modeling of Routinely Collected Data to Assess the Effect of Health Programs in Malaria Incidence During Pregnancy in Burkina Faso
Source: Sci Rep. 2020 Feb 14;10:2618. doi: 10.1038/s41598-020-58899-3 (PMC7021681; doi:10.1038/s41598-020-58899-3)
Supplement: Supplementary file 8 — Supplementary information8 [file 41598_2020_58899_MOESM8_ESM.pdf]

Bayesian Spatiotemporal Modeling of Routinely Collected Data to Assess the Effect of Health Programs in Malaria Incidence During Pregnancy in Burkina Faso

Toussaint Rouamba[1,2], Sekou Samadoulougou[3,4], Halidou Tinto[1], Victor A. Alegana[5,6], and Fati Kirakoya–Samadoulougou[2]

1 Clinical Research Unit of Nanoro, Institute for Research in Health Sciences, National Center for Scientific and Technological Research, 528, Avenue Kumda-Yoore, BP 218 Ouagadougou CMS 11, Ouagadougou, Burkina Faso
2 Center for research in epidemiology, Biostatistics and Clinical Research, School of Public Health, University libre de Bruxelles (ULB), Route de Lennik, 808 B-1070 Bruxelles. Brussels, Belgium
3 Centre for Research on Planning and Development (CRAD), Laval University, Quebec, G1V 0A6, Canada
4 Evaluation Platform on Obesity Prevention, Quebec Heart and Lung Institute, Quebec, G1V 4G5, Canada.
5.Kenya Medical Research Institute - Wellcome Trust Research Programme, P.O. Box 43640-00100, Nairobi, Kenya
6. Geography and Environmental Science, University of Southampton, SO17 1BJ Southampton, UK

*Correspondence to rouambatoussaint@gmail.com

Supplementary Information 8

Figure S8. Maps showing the monthly spatio-temporal dynamic in term of space-time interaction (Y_{kt}) of malaria rates in each community from 2015 to 2017
Source: The shapefile was obtained from the "Base Nationale de Découpage du territoire" of Burkina Faso (BNDT, 2006). The malaria in pregnancy data were obtained from the National Malaria Control Program of Burkina Faso, downloaded from the District Health Information System 2.
Maps created by Toussaint Rouamba et al, 2019

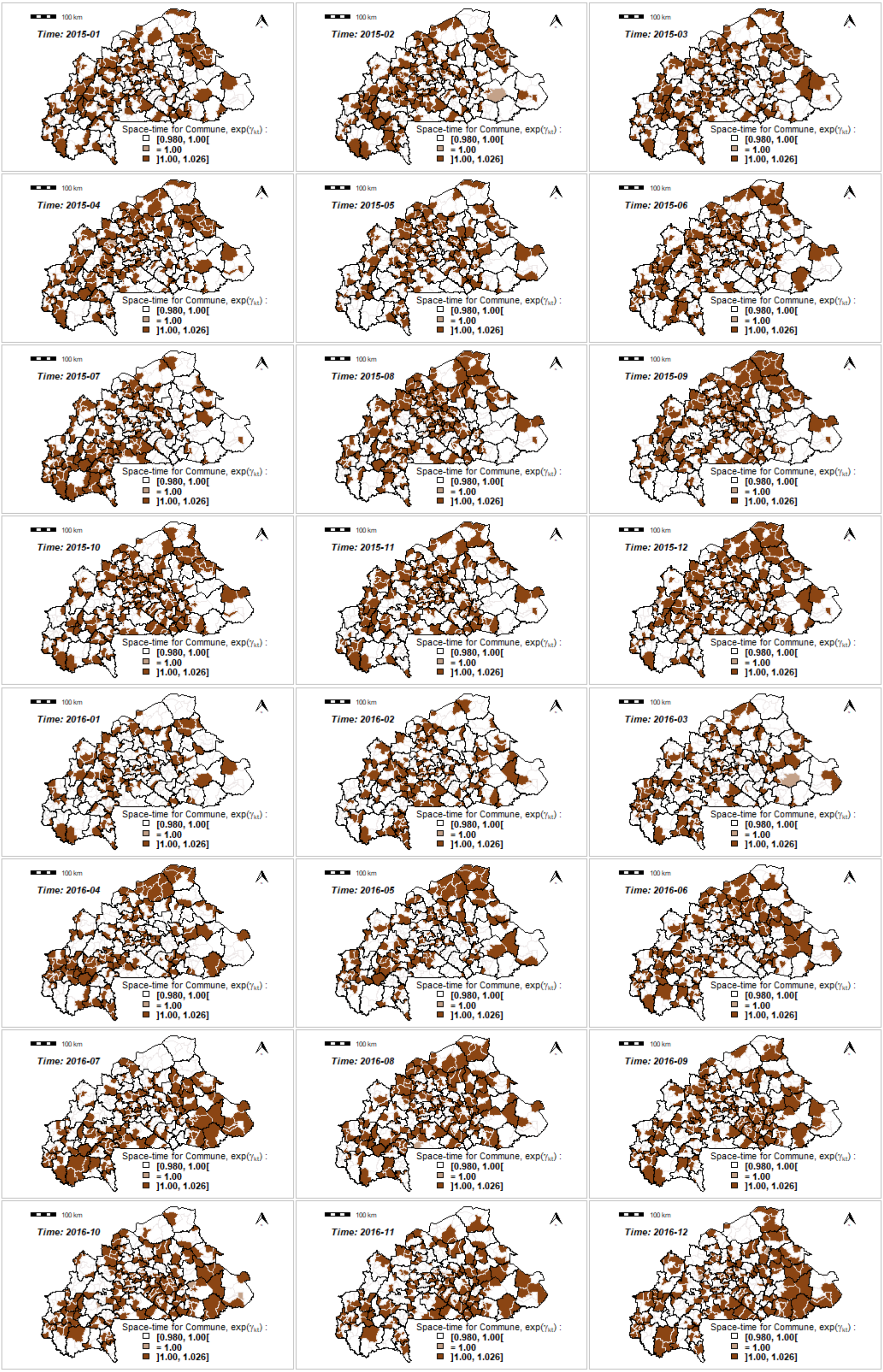

100 km
Time: 2015-01
Time: 2015-02
Time: 2015-03
Time: 2015-04
Time: 2015-05
Time: 2015-06
Time: 2015-07
Time: 2015-08
Time: 2015-09
Time: 2015-10
Time: 2015-11
Time: 2015-12
Time: 2016-01
Time: 2016-02
Time: 2016-03
Time: 2016-04
Time: 2016-05
Time: 2016-06
Time: 2016-07
Time: 2016-08
Time: 2016-09
Time: 2016-10
Time: 2016-11
Time: 2016-12
Space-time for Commune, exp(γ_{kt}) :
[0.980, 1.00[
= 1.00
]1.00, 1.026]

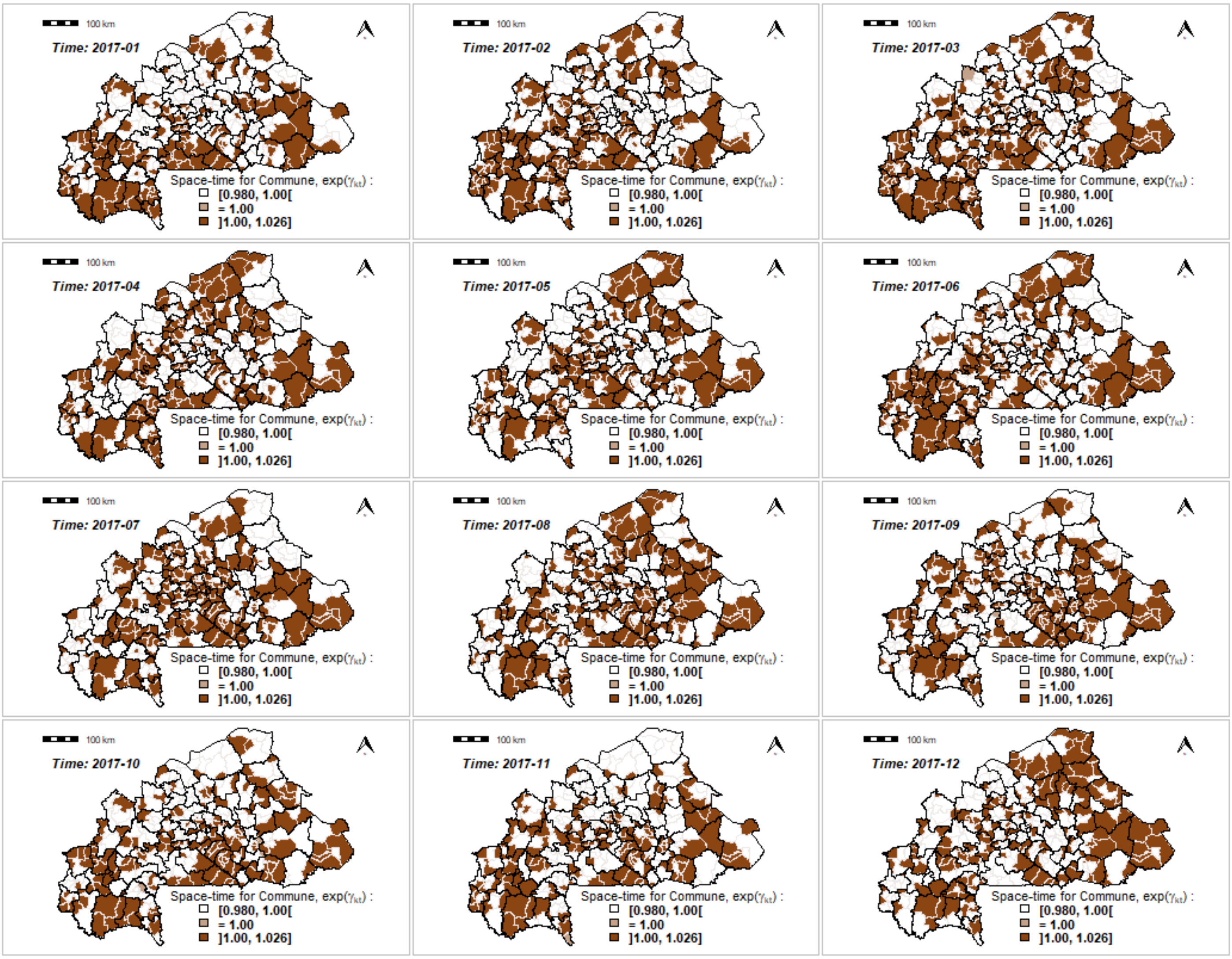
100 km
Time: 2017-01
Space-time for Commune, exp(γ_{kt}) :
[0.980, 1.00[
= 1.00
]1.00, 1.026]
100 km
Time: 2017-02
Space-time for Commune, exp(γ_{kt}) :
[0.980, 1.00[
= 1.00
]1.00, 1.026]
100 km
Time: 2017-03
Space-time for Commune, exp(γ_{kt}) :
[0.980, 1.00[
= 1.00
]1.00, 1.026]
100 km
Time: 2017-04
Space-time for Commune, exp(γ_{kt}) :
[0.980, 1.00[
= 1.00
]1.00, 1.026]
100 km
Time: 2017-05
Space-time for Commune, exp(γ_{kt}) :
[0.980, 1.00[
= 1.00
]1.00, 1.026]
100 km
Time: 2017-06
Space-time for Commune, exp(γ_{kt}) :
[0.980, 1.00[
= 1.00
]1.00, 1.026]
100 km
Time: 2017-07
Space-time for Commune, exp(γ_{kt}) :
[0.980, 1.00[
= 1.00
]1.00, 1.026]
100 km
Time: 2017-08
Space-time for Commune, exp(γ_{kt}) :
[0.980, 1.00[
= 1.00
]1.00, 1.026]
100 km
Time: 2017-09
Space-time for Commune, exp(γ_{kt}) :
[0.980, 1.00[
= 1.00
]1.00, 1.026]
100 km
Time: 2017-10
Space-time for Commune, exp(γ_{kt}) :
[0.980, 1.00[
= 1.00
]1.00, 1.026]
100 km
Time: 2017-11
Space-time for Commune, exp(γ_{kt}) :
[0.980, 1.00[
= 1.00
]1.00, 1.026]
100 km
Time: 2017-12
Space-time for Commune, exp(γ_{kt}) :
[0.980, 1.00[
= 1.00
]1.00, 1.026]
